# High-Precision DOA Estimation Based on Synthetic Aperture and Sparse Reconstruction

**DOI:** 10.3390/s23218690

**Published:** 2023-10-24

**Authors:** Yang Fang, Xiaolong Wei, Jianjun Ma

**Affiliations:** 1 State Key Laboratory for Strength and Vibration of Mechanical Structures, Shaanxi Engineering Research Centre of NDT and Structural Integrity Evaluation, School of Aerospace Engineering, Xi’an Jiaotong University, Xi’an 710049, China; 2 Science and Technology on Plasma Dynamics Laboratory, Air Force Engineering University, Xi’an 710000, China; wei18892022001@163.com; 3 National Laboratory of Radar Signal Processing, Xidian University, Xi’an 710071, China; mjj120911@163.com

**Keywords:** direction-of-arrival (DOA) estimation, synthesis array, compressed sensing (CS), Bayesian frame

## Abstract

The direction-of-arrival (DOA) estimation is predominantly influenced by the antenna’s aperture size. However, space constraints on flight platforms often necessitate the use of antennas with smaller apertures and fewer array elements. This inevitably imposes limitations on the DOA estimation’s resolution and degrees of freedom. To address these precision constraints, we introduce an accurate DOA estimation method based on spatial synthetic aperture model. This method adopts a two-stage strategy to ensure both efficiency and precision in DOA estimation. Initially, the orthogonal matching pursuit (OMP) reconstruction algorithm processes the original aperture data, providing a rough estimate of target angles that guides the aircraft’s flight direction. Subsequently, the early estimations merge with the aircraft’s motion space samples, forming equivalent spatially synthesized array samples. The refined angle estimation then employs the OMP-RELAX algorithm. Moreover, with the off-grid issue in mind, we devise an estimation method integrating Bayesian parameter estimation with dictionary sequence refinement. The proposed technique harnesses the spatial synthetic aperture for pinpoint estimation, effectively addressing the challenges of atomic orthogonality and angular off-grid on estimation accuracy. Importantly, the efficiency of deploying sparse reconstruction for angle estimation is bolstered by our phased strategy, eliminating the necessity for fine grid analysis across the entire observation scene. Moreover, the poor estimation accuracy caused by coherent source targets and angular-flickering targets is improved by sparse reconstruction. Through simulation and experiment, we affirm the proposed method’s efficacy in angle estimation. The results indicate that target angle estimation errors are limited to within 1°. Furthermore, we assess the impact of variables such as target state, heading angle, spatial sampling points, and target distance on the estimation accuracy of our method, showcasing its resilience and adaptability.

## 1. Introduction

Enhancing the Direction of Arrival (DOA) estimation is one of the ways to improve radar detection accuracy [1,2]. However, the spatial constraints on the platform mean that only small aperture radars can be accommodated. With radar beam-width inversely related to antenna aperture, achieving precise angles via traditional DOA estimation techniques poses challenges [3,4,5]. This tension between the demand for precise DOA estimation—especially for coherent source DOA—and radar resource constraints underscores the limitations of current DOA methods. Angle glint is another obstacle: echo power may diminish at certain angles or times due to adversarial radar interference. This makes angle estimation particularly challenging. Thus, there is an urgent need to investigate high-resolution DOA estimation methods adaptable to radar constraints. Synthetic aperture techniques for DOA estimation, which may offer improved accuracy, present an intriguing area of research.

Numerous DOA estimation methods have emerged over the past few decades. Spatial spectrum estimation techniques such as MUSIC [6,7,8] and ESPRIT [9,10] are commonly utilized for incoherent source angle estimation. These methods depend on statistical analyses, harnessing covariance from multiple snapshots and employing eigenvalue analysis to realize super-resolution DOA estimation. However, for coherent sources, steer vectors often converge to the same eigenvalue subspace, rendering this method less effective [11,12,13]. Additionally, these methods suffer from low efficiency due to eigenvalue decomposition, making them ill-suited for time-sensitive DOA estimation [14,15]. Alternative techniques like the iterative adaptive (IAA) method [16] have been suggested. These methods utilize weighted least squares (WLS) for super-resolution spectral estimation and the Toeplitz-FFT method to improve computational efficiency [17,18]. However, even with Toeplitz-FFT integration, they might not adequately meet the efficiency standards for radar [19]. For coherent source DOA estimation, temporal smoothing techniques combined with MUSIC and ESPRIT have been proposed [20,21]. In reference [22], a dual-polarization synthetic sparse nested array consisting of three subarrays is proposed. Based on the sparse nested array, a DOA estimation method is proposed to improve the estimation accuracy, which is first rough estimation and then precise estimation. Recently, methods harnessing compressive sensing have been advanced for both coherent and incoherent DOA estimation [23,24]. These methods capitalize on sparsity in the angle domain, with [25] integrating sequential snapshots with sparsity priors for Bayesian inference-based sequential DOA estimation, and [26,27] applying multiple snapshots to devise the MMV-OMP sparsity reconstruction method for coherent DOA estimation. Although these methods can provide super-resolution estimation, they may experience performance degradation in radar applications due to the small antenna aperture.

To address these challenges, this paper introduces a two-stage DOA estimation technique. Initially, the original aperture facilitates a rough angle estimation, which then determines the refinement measurement grid and sets the flight trajectory. Subsequently, merging the initial target angles with spatial samples from the moving target, these samples are treated as equivalent spatial synthetic array samples. A measurement dictionary, rooted in spatial samples, is established, and sparse reconstruction is applied for angle estimation. To bolster DOA estimation accuracy, a sequential estimation method, amalgamating Bayesian proportion parameter estimation with dictionary refinement, is designed. The method presented holds three distinct advantages:

(1) Enhanced Precision: The spatial domain’s synthetic array, with its expanded aperture and heightened resolution, combined with sequential estimation that integrates Bayesian proportion parameter estimation and dictionary refinement, delivers a more precise DOA.
(2) Augmented Efficiency: Rough angle estimation results serve as the foundation for subsequent estimations, significantly diminishing the algorithm’s computational complexity. And the DOA estimation process uses the greedy method of sparsity reconstruction, catering to the need for rapid estimations.
(3) Mitigation of angle glint issues: The spatial synthesis array sample and target angle reconstruction can be considered equivalent to a spatial matched filter. Even though the target’s power may be weak over a certain period, DOA estimation can still be accomplished using spatial accumulation over an extended duration.

The remainder of this paper is organized as follows: Section 2 outlines the signal model and method, while Section 3 discusses comparative tests and performance analysis. Lastly, conclusions on the proposed method are summarized in the final section.

## 2. Method Description

### 2.1. Synthesis Array DOA Estimation Signal Model

**Figure 1:**
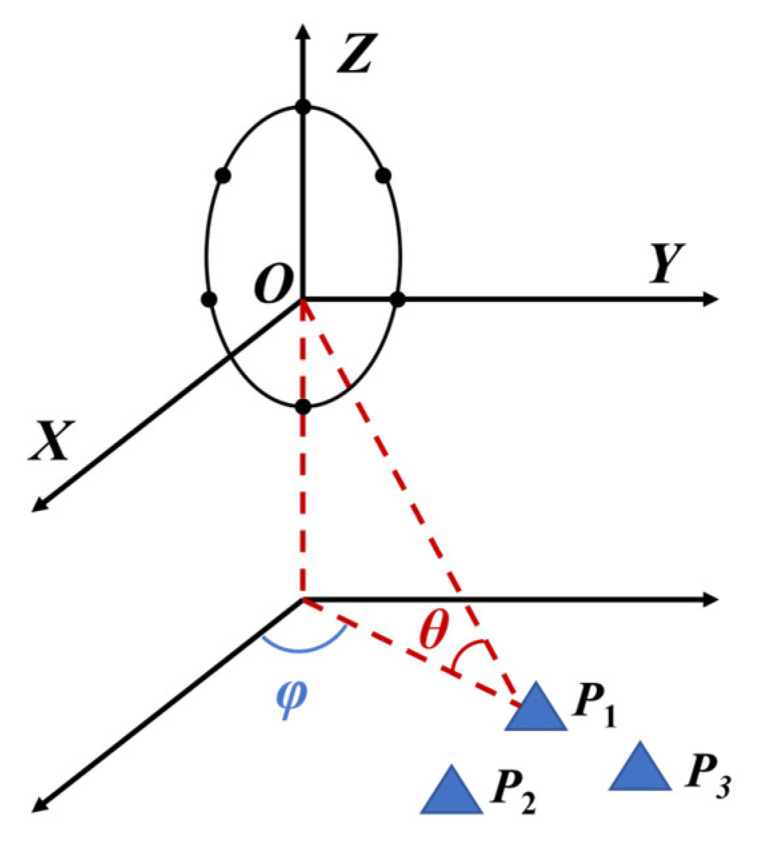
Radar target space geometry.

We assume that our radar system is tasked with estimating three coherent sources, as depicted in Figure 1. To facilitate this, a uniform circular array is incorporated into the radar system. The origin point signifies the initial measurement position, and the axis direction is illustrated in Figure 1. The azimuth angle φ is the angle formed between the target and the *X*-axis. Concurrently, the pitch angle θ represents the angle between the target and the XOY plane. Targets are configured in an equilateral triangle and are labeled as $P_{1}$, $P_{2}$, and $P_{3}$, with their respective coordinates and angles designated as $P_{1}(x_{1},y_{1},z_{1})$,$P_{2}(x_{2},y_{2},z_{2})$,$P_{3}(x_{3},y_{3},z_{3})$ and $P_{1}(\varphi_{1},\theta_{1})$,$P_{2}(\varphi_{2},\theta_{2})$,$P_{3}(\varphi_{3},\theta_{3})$. respectively.

The coordinates of the three targets relate as follows:

(1) $$\begin{array}{l} {\left[x_{2}-x_{1}\;y_{2}-y_{1}\right]}^{T}=\Phi {\left[x_{3}-x_{1}\;y_{3}-y_{1}\right]}^{T}\\ {\left[x_{3}-x_{2}\;y_{3}-y_{2}\right]}^{T}=\Phi {\left[x_{3}-x_{1}\;y_{3}-y_{1}\right]}^{T} \end{array},$$

where Φ is the rotation matrix:

(2) $$\Phi =\left[\begin{array}{l} \cos(60)\;-\sin(60)\\ \sin(60)\;\cos(60) \end{array}\right].$$

Angles can be deduced from the target’s coordinates as

(3) $$\theta_{i}=\arctan(\frac{x_{i}}{y_{i}}),\;\varphi_{i}=\arctan(\frac{z_{i}}{\sqrt{x_{i}^{2}+y_{i}^{2}}}),\;i=1,2,3.$$

Let us denote the target steering vector as $a(\varphi_{i},\theta_{i})$ and the signal envelope as s(t). If we assume noise n(t) in every snapshot to be independent and identically distributed Gaussian white noise with a noise power $\sigma^{2}$, the resultant signal can be expressed as

(4) $$y(t)=\sum_{i=1}^{I}A_{i}a(\varphi_{i},\theta_{i})s_{i}(t)+n(t),$$

where $A_{i}$ is the reflection coefficient of the ith target, and ***y***(*t*) denotes all the reflected signals in the observation scene received by the antennas.

**Figure 2:**
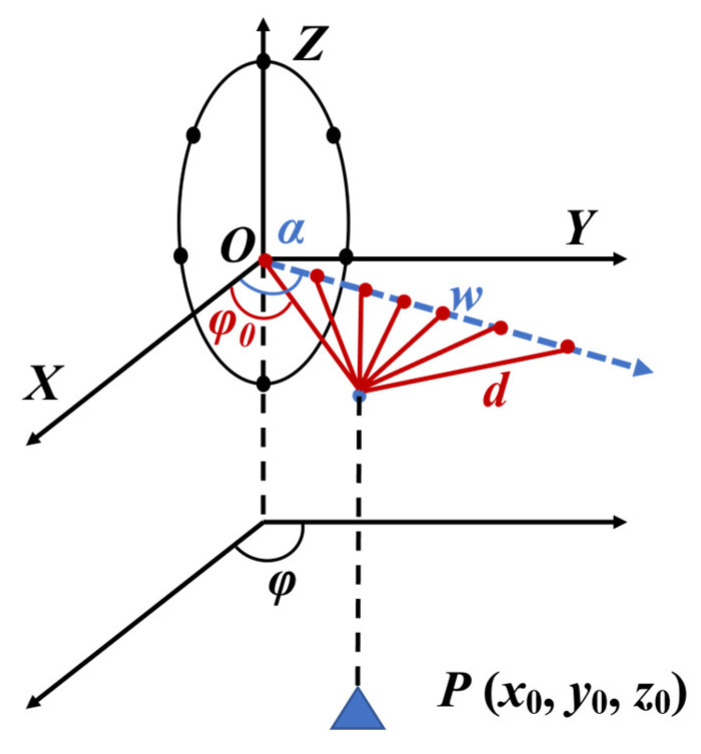
Spatial synthetic aperture geometry.

To enlarge the array aperture, the movement of the platform is employed to design the spatial synthesis array, depicted in Figure 2.

We suppose the platform’s heading angle is α, its velocity is v, and the spatial sampling time is $T_{sp}$. If the target’s initial azimuth is $\varphi_{0}$ and its coordinates are given by $p(x_{0},y_{0},z_{0})$, the vertical distance from the target to the airline can be expressed as

(5) $$d=\frac{\left|y_{0}-\tan(\alpha -\varphi_{0})x_{0}\right|}{\sqrt{1+\tan^{2}(\alpha -\varphi_{0})}}.$$

The center point of the scene is defined as the projection of the target in the flight direction, so distance w between scene center $o^{\prime }$ and the platform is

(6) $$w=\frac{d}{\tan(\alpha -\varphi )}.$$

Consequently, the distance from the ith spatial sample to the scene center is

(7) $$l_{i}=\sqrt{{\left(w-iT_{sp}v\right)}^{2}+d^{2}+h^{2}},$$

where h is the elevation of the platform from the ground. The phase difference from the ith spatial sample of the platform to the original spatial sample is

(8) $$\Delta_{i}=\frac{2\pi }{\lambda }(l_{i}-l_{0}).$$

Under these conditions, the comprehensive spatial sample steering vector becomes

(9) $$a_{sp}={\left[\exp(j\Delta_{0}),\exp(j\Delta_{1})\ldots \exp(j\Delta_{M})\right]}^{T}.$$

After accumulating *M* spatial samples, the spatial echo across arrays can be described as

(10) $$y_{sp}=a_{sp}⊙{\left[s(0•T_{sp}),s(1•T_{sp})\ldots s(M•T_{sp})\right]}^{T},$$

$$y_{sp}=a_{sp}⊙[s\left(0\cdot T_{sp}\right),s\left(1\cdot T_{sp}\right),\ldots ,s\left(M\cdot T_{sp}\right)]$$

where ${\left[a_{1},a_{2}\ldots a_{M}\right]}^{T}⊙{\left[b_{1},b_{2}\ldots b_{M}\right]}^{T}={\left[{a_{1}b}_{1},{a_{2}b}_{2},\ldots {,a_{M}b}_{M}\right]}^{T}$${\left[a_{1},a_{2},\ldots a_{M}\right]}^{T}⊙{\left[b_{1},b_{2},\ldots b_{M}\right]}^{T}={\left[a_{1}b_{1},a_{2}b_{2},\ldots a_{M}b_{M}\right]}^{T}$, the radar equivalent aperture is $L=vMT_{sp}$. With an adequate number of samples, the synthesized beam width is sufficiently narrow to discern target angles. 

### 2.2. Rough DOA Estimation

Initially, we employ the maximum coherence method to provide a rough estimation of target angles using the original radar array. The angle dictionary is formulated as

(11) $$A(\varphi ,\theta )=[a(\varphi_{j},\theta_{i})],i=1,2\ldots .I,j=1,2\ldots .J,$$

where A consists of spatial steering vectors, and the angle grid is partitioned according to the beam width. Given that multiple targets fall within a single beam, the maximum coherence between several snapshots and the angle dictionary can be harnessed to ascertain the rough angle. This estimation can be represented by the following equation:

(12) $$[{\hat{\varphi }}_{rough},{\hat{\theta }}_{rough}]=mean(\underset{\left(\varphi_{t},\theta_{t}\right)}{\max}\left|s{\left(t\right)}^{T}A(\varphi ,\theta )\right|),t=0,T_{s},2T_{s}\ldots .,$$

where $T_{s}$ denotes the snapshot sampling time. Each snapshot provides a rough angle estimation through the metric of maximum coherence. At this time, the angle grid in the dictionary atom can be divided according to the beam width of the array. The estimation of the rough angle can be used to plan the flight direction of the aircraft. Due to the limitation of the aperture, when the angle interval of the target is too small, it is unable to distinguish multiple targets, so the rough estimation only needs to obtain the approximate angle of the target. By averaging across numerous snapshots, a more refined rough estimation is acquired. Subsequently, to differentiate between targets, the spatial sample synthesis array is leveraged to expand the radar’s aperture, facilitating a more accurate angle estimation.

### 2.3. Refined DOA Estimation

In order to both minimize the count of spatial samples and enhance the efficiency of angle estimation, a sparse reconstruction framework is proposed. This serves as an effective method for super-resolution estimation of target angles. Let us denote the precise angle grid as $\Delta \varphi_{precious}$ and $\Delta \theta_{precious}$. (It is worth noting that the angle division here builds upon the preceding step, utilizing the roughly estimated angle from that phase as the foundation for this more refined estimation.) The complete spatial domain’s precise steering vector, denoted as $A_{precious}$, is derived; refer to Equations (5)–(9). Consequently, our signal model can be expressed as

(13) $$y_{sp}=A_{precious}x+n,$$

where $A_{precious}=[a(\Delta \varphi_{precious,i},\Delta \theta_{precious,j})]$, and x stands for the echo intensity vector associated with a given angle. x is sparse, signifying that the majority of its elements are zero, with a much smaller number being non-zero. As such, x can be estimated via the following optimization function:

(14) $$\min{\left\|y_{sp}-A_{precious}x\right\|}_{2}^{2},s.t.{\left\|x\right\|}_{0}<\varepsilon .$$

This presents a non-convex challenge. However, it can be addressed through greedy algorithms like MP (Matching Pursuit) and OMP (Orthogonal Matching Pursuit). To incorporate multiple snapshot data, the MMV-OMP (Multiple Measurement Vector-OMP) approach can be employed to resolve Equation (14).

### 2.4. Dynamic Off-Grid Estimation Based on Bayesian Theory

The method previously discussed allows for more accurate estimation of adjacent target angles. However, with spatial sampling and estimation, two pressing issues remain:

(1) Off-Grid Estimation: Our dictionary dictates that angles can only be estimated based on its specific grid. Unfortunately, actual angles often deviate from this grid, leading to potential errors.
(2) Non-Orthogonal Atoms: We utilize the OMP method for angle estimation, but it is crucial to recognize that the atoms in our angle dictionary are not orthogonal. Given that amplitude estimation relies on orthogonal projection, the estimated angle typically falls between two adjacent atoms.

Beyond post-sampling estimation, a dynamic estimation process could be crafted to conduct spatial sampling and angle estimation concurrently. This real-time process can utilize error information as it emerges, enhancing estimation precision. To tackle these issues and refine precision, we propose a DOA estimation method based on sequential off-grid Bayesian reference, which refines the measurement dictionary.

To address non-orthogonal atoms, the OMP-RELAX method offers a potential solution to boost estimation performance [28]. The RELAX method, a super-resolution spectral estimation technique, uses iterative re-projection to diminish atom correlation introduced by non-orthogonal atom side-lobes. Thus, RELAX is deployed post each OMP estimation.

For the off-grid challenge, we present a dynamic Bayesian off-grid method, integrated with sequential sampling. As spatial sampling grows, so does angle estimation resolution. Given there are N samples during kth estimation, re-estimating the angle post each $N_{T}$ sample becomes feasible. Focusing on the azimuth angle synthesis aperture, the azimuth beam-width during kth estimation can be gauged as

(15) $$\varphi_{bw}=\frac{1}{\cos(\varphi_{0})}\frac{50.8\lambda }{NvT_{sp}},$$

where $\varphi_{0}$ defines the target azimuth, and λ signifies the wavelength. From this, we can derive the beam width during the (k+1)th estimation and delineate the angle grid of the dictionary for subsequent estimations using the beam width. The angle can be re-estimated after each $N_{T}$ sample, and the beam width can be calculated during the (k+1)th estimation as

(16) $$\Delta \varphi_{k+1}=\frac{1}{R}•\frac{1}{\cos(\varphi_{0})}\frac{50.8\lambda }{(N+N_{T})vT_{sp}}.$$

$${∆\varphi }_{k+1}=\frac{1}{R}\cdot \frac{1}{\cos\left(\varphi_{0}\right)}\frac{50.8\lambda }{(N+N_{T})vT_{sp}}$$

Considering the kth estimation, the measurement dictionary can be refined according to the resolution near the angle ${\hat{x}}_{k}$. Combining the angle resolution from the aforementioned equation, the real angle lies within

(17) $$x\in [{\hat{x}}_{k}-\frac{\Delta \varphi_{k}}{2},{\tilde{x}}_{k+1}+\frac{\Delta \varphi_{k+1}}{2}].$$

We posit ${\hat{x}}_{k}<{\tilde{x}}_{k+1}$ and introduce proportion parameters α(0≤α≤1) to describe the real angle as

(18) $$x=\alpha •({\hat{x}}_{k}-\frac{\Delta \varphi_{k}}{2})+(1-\alpha )•({\tilde{x}}_{k+1}+\frac{\Delta \varphi_{k+1}}{2}).$$

$$x=\alpha \cdot \left({\hat{x}}_{k}-\frac{∆\varphi_{k}}{2}\right)+(1-\alpha )\cdot ({\tilde{x}}_{k+1}+\frac{∆\varphi_{k+1}}{2})$$

It is pertinent to note that the real angle measurement atom evolves as a function of the angle from Equations (5)–(9). Using the first-order Taylor approximation of Equation (5), we can express

(19) $$A(x)=A({\tilde{x}}_{k+1})+A\prime ({\tilde{x}}_{k+1})(x-{\tilde{x}}_{k+1})+O(x-{\tilde{x}}_{k+1}).$$

Integrating the above into the derived Equation (18) and overlooking higher-order terms, the atom becomes

(20) $$A(x)\approx [A({\tilde{x}}_{k+1})+\frac{\Delta \varphi_{k+1}}{2}A\prime ({\tilde{x}}_{k+1})]+\alpha •[A\prime ({\tilde{x}}_{k+1})({\hat{x}}_{k}-{\tilde{x}}_{k+1}-\frac{\Delta \varphi_{k+1}}{2}-\frac{\Delta \varphi_{k}}{2})].$$

$$A\left(x\right)\approx \left[A\left({\tilde{x}}_{k+1}\right)+\frac{∆\varphi_{k+1}}{2}A^{\prime }\left({\tilde{x}}_{k+1}\right)\right]+\alpha \left[A^{\prime }\left({\tilde{x}}_{k+1}\right)({\hat{x}}_{k}-{\tilde{x}}_{k+1}-\frac{∆\varphi_{k+1}}{2}-\frac{∆\varphi_{k}}{2})\right]$$

Estimating α enables pinpointing the exact angle. The subsequent section provides the estimation of α within the Bayesian context. 

The relationship between the measurement signal and real parameters in the (k+1)th spatial sampling can be expressed as

(21) $$y_{k+1}=A(x)x_{x}+n.$$

Substituting Equation (20) into Equation (21) results in an accurate measurement model based on estimation parameters, as follows:

(22) $$y_{k+1}=[P({\tilde{x}}_{k+1})+\alpha •Q({\tilde{x}}_{k+1},{\hat{x}}_{k})]x_{x}+n,$$

$$y_{k+1}=\left[P\left({\tilde{x}}_{k+1}\right)+\alpha \cdot Q({\tilde{x}}_{k+1},{\hat{x}}_{k})\right]x_{x}+n$$

where

(23) $$\left\{\begin{array}{l} P({\tilde{x}}_{k+1})=[A({\tilde{x}}_{k+1})+\frac{\Delta \varphi_{k+1}}{2}A\prime ({\tilde{x}}_{k+1})]\\ Q({\tilde{x}}_{k+1},{\hat{x}}_{k})=[A\prime ({\tilde{x}}_{k+1})({\hat{x}}_{k}-{\tilde{x}}_{k+1}-\frac{\Delta \varphi_{k+1}}{2}-\frac{\Delta \varphi_{k}}{2})] \end{array}\right..$$

Assuming the noise is Gaussian white noise, the probability distribution is $n\sim N(0,\sigma^{2}{}_{n})$, and the amplitude distribution is $x_{x}\sim N(x_{{\tilde{x}}_{k+1}},\sigma^{2}{}_{x})$, where $x_{{\tilde{x}}_{k+1}}$ is the estimated amplitude after OMP-RELAX refined estimation. Consequently, the posterior distribution of amplitude $x_{x}$ can be represented by a complex Gaussian using Bayesian formulation as

(24) $$p(x_{x}|y_{k+1};\sigma_{n},\sigma_{x})=\frac{p(y_{k+1}|x_{x};\sigma_{n},\sigma_{x})p(x_{x}|\sigma_{x})}{\int p(y_{k+1}|x_{x};\sigma_{n},\sigma_{x})p(x_{x}|\sigma_{x})dx_{x}}.$$

The complex Gaussian distribution is $p(x_{x}|y_{k+1},\sigma_{n},\sigma_{x})\sim CN(\mu_{k+1},\Sigma_{k+1})$, where

(25) $$\left\{\begin{array}{l} \mu_{k+1}=\Sigma_{k+1}(\beta A{\left(x\right)}^{H}y_{k+1}+\Sigma_{x}{}^{-1}{\tilde{x}}_{k+1})\\ \Sigma_{k+1}={\left(\beta A{\left(x\right)}^{H}A(x)+\Sigma_{x}{}^{-1}\right)}^{-1} \end{array}\right..$$

Referencing [29,30], the proportion parameter α can be estimated using the maximum posterior with the EM iteration method. In the E-step, parameter $x_{x}$ is treated as a hidden variable, and given the fixed parameters $y_{k+1}$, $\sigma_{n}$, and $\sigma_{x}$ in the ith iteration, the E-step expectation can be presented as

(26) $$E_{x_{x}|y_{k+1};\sigma_{n}^{\left(i\right)},\sigma_{x}^{\left(i\right)}}[\ln(p(y_{k+1},x_{x}\;|\;\sigma_{n},\sigma_{x}))],$$

where $p(y_{k+1},x_{x}|\sigma_{n},\sigma_{x})$ can be deduced using Bayesian formulation as

(27) $$p(y_{k+1},x_{x}\;|\;\sigma_{n},\sigma_{x})=p(y_{k+1}\;|\;x_{x};\sigma_{n},\sigma_{x})p(x_{x}\;|\;\sigma_{x}).$$

In this case, Equation (26) can be calculated. The next step, the M-step, involves estimating the parameters by maximizing Expectation (26):

(28) $$\sigma_{n}{}^{i+1},\sigma_{x}{}^{i+1}=\underset{\sigma_{n},\sigma_{x}}{\arg\max}E_{x_{x}\;|\;y_{k+1};\sigma_{n}^{\left(i\right)},\sigma_{x}^{\left(i\right)}}[\ln(p(y_{k+1},x_{x}\;|\;\sigma_{n},\sigma_{x}))].$$

Following a similar approach as in [31], the proportion parameter α can be estimated concurrently with $\sigma_{x}{}^{i+1}$, and the amplitude estimation can use the expectation of the posterior distribution, that is,

(29) $$\left\{\begin{array}{l} {\hat{\alpha }}_{l}^{i+1}=\frac{ℜ(\mu_{l}{}^{*}q_{l}{}^{H}(y_{k+1}-P\mu ))-ℜ(q_{l}{}^{H}P\gamma_{i})}{{\left\|p_{l}\right\|}_{2}^{2}\mu_{l}+\gamma_{i,i}{\left\|p_{l}\right\|}_{2}^{2}}\\ {\hat{x}}_{x}{}^{i+1}=\mu^{i+1} \end{array}\right..$$

After several iterations, proportion parameter α converges near the real value, allowing the estimation in the (k+1)th spatial sampling to be expressed as

(30) $${\hat{x}}_{{}_{k+1}}={\hat{\alpha }}_{k+1}{\hat{x}}_{k}+(1-{\hat{\alpha }}_{k+1}){\tilde{x}}_{k+1}.$$

### 2.5. Overall Procedure of the Proposed Method

**Figure 3:**
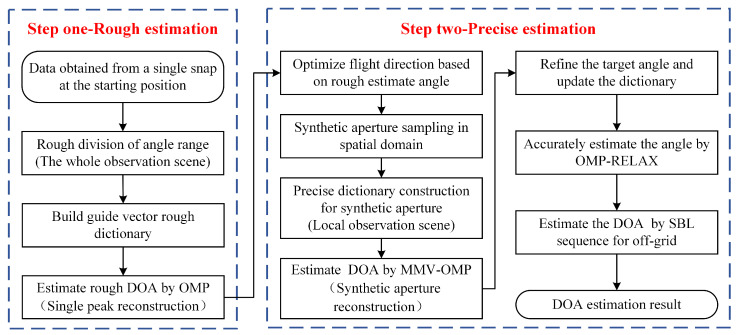
Diagram representing the flow of the proposed algorithm.

Figure 3 illustrates the comprehensive flow of the proposed method, which can be broken down into four main steps:

Step 1: Initial Angle Estimation using a Single Snapshot Signal

- Initially, using the beam width, the angle interval is coarsely divided.
- The dictionary matrix is then assembled using Equation (11).
- For a singular snapshot dataset, the OMP algorithm is used to solve Equation (12) for singular peak reconstruction, which provides the primary rough estimation of the target angle.

Step 2: Angle Estimation via the Synthesized Array Signal

- Based on the previously estimated result $\left[{\hat{\varphi }}_{rough},{\hat{\theta }}_{rough}\right]$, the aircraft’s heading angle α is planned.
- The number Q of sampling points is predetermined.
- Leveraging multiple snapshot datasets, a locally refined angle comprehensive guide vector matrix $A_{precious}$ is fashioned using Equations (5)–(9).
- The MMV-OMP algorithm is then deployed to provide a rough angle estimation.

Step 3: Correction for Non-Orthogonal Dictionary Atomic Errors

- The OMP-RELAX algorithm rectifies errors stemming from non-orthogonal atoms Φ.
- The angle estimation derived from the previous stage serves as the input.
- The angle undergoes refinement by $\Phi_{refine}=\left(\Phi -\delta ,\Phi -\delta +\frac{\left(\Delta \varphi ,\Delta \theta \right)}{\eta },\cdots ,\Phi +\delta \right)$, where Δφ and Δθ correspond to the azimuth and elevation angle interval divisions during the initial estimation, respectively.
- The refinement parameter $\eta =(\eta_{\varphi },\eta_{\theta })$ is determined based on the desired resolution precision.
- The angle constraint $\delta =(\delta_{\varphi },\delta_{\theta })$ is set in accordance with the current angle interval division, typically half of the present angle interval.
- Utilizing this, the revised dictionary matrix $A(\Phi_{refine})$ is acquired. Subsequently, the OMP algorithm procures K revised target angle estimations.

Step 4: Off-Grid Rectification

- Drawing from the previous step’s estimated value and coupled with spatial sequence sampling, the sequential Bayesian dynamic estimation technique is employed to address positional deviations due to off-grid anomalies.
- The initial values $\left(\alpha_{0},\sigma_{n_{0}},\sigma_{x_{0}}\right)$ of the undisclosed parameter are set, with the iteration number fixed at K.
- Using Equation (28), the matrices $\Sigma_{k+1}$ and $\mu_{k+1}$ are refreshed.
- Equations (29) and (30) facilitate updates to $\left(\sigma_{n_{0}},\sigma_{x_{0}}\right)$ and $\alpha_{0}$, respectively.
- Through iterative solutions, the final precise target angle estimation is derived.

## 3. Simulation and Experimental Verification and Analysis

### 3.1. Validation of Effectiveness

To verify the effectiveness of the method proposed in this paper, simulation data were used. The radar and target scene settings are depicted in Figure 2. The circular array is made up of seven omnidirectional array elements with an array radius of 8 cm. The radar emits a linear frequency-modulated signal at a carrier frequency of 3.75 GHz. The carrier’s altitude is 1.5 km, the distance from the radar to the scene’s center is 5 km, and the spatial sampling interval measures 0.04 m. The angular coordinates of the three targets in the scene are $P_{1}(20.1{}^{\circ},-17.4{}^{\circ})$, $P_{2}(23.7{}^{\circ},-16.2{}^{\circ})$, and $P_{3}(26.1{}^{\circ},-17.6{}^{\circ})$.

**Figure 4:**
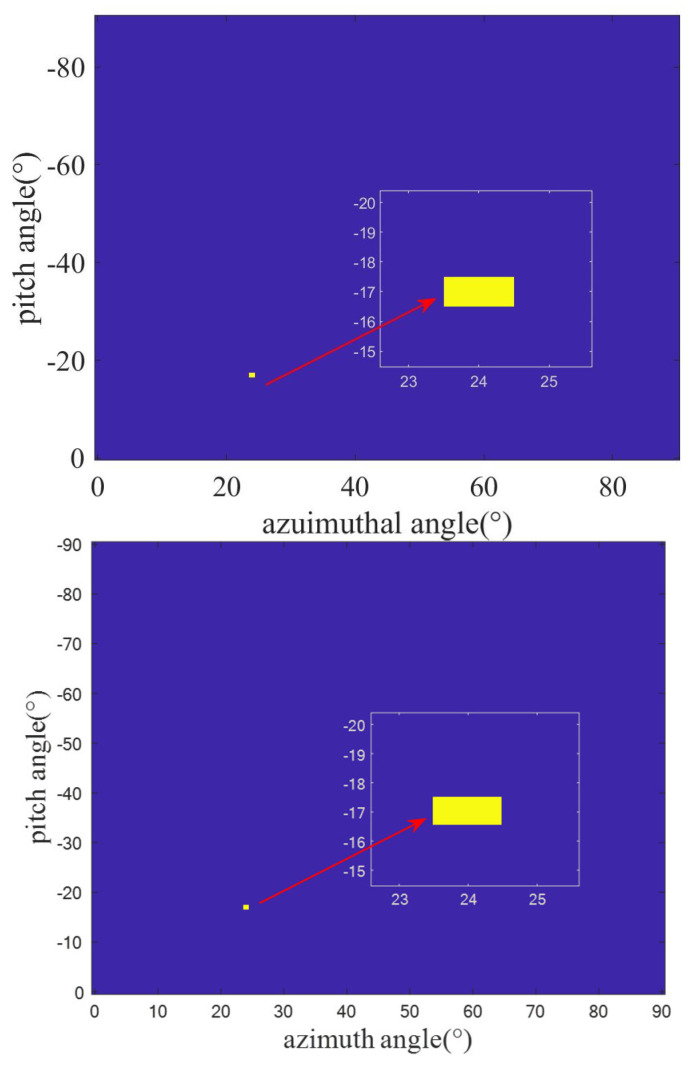
Initial rough estimation result. (The area pointed by the red arrow in the figure is an enlargement of the target area.)

Given the proximity of the targets, their azimuth and elevation angles are notably similar. During the initial rough estimation, both azimuth and elevation intervals are set at 1°, with search ranges designated at (0, 90°) for the azimuth and at (−90°, 0°) for elevation angles. Figure 4 presents the initial rough estimation result, highlighting an estimated azimuth angle of 24° and an elevation angle of −17°. The area pointed by the red arrow in the figure is an enlargement of the target area. Due to the pronounced correlation among adjacent atoms in the dictionary (with a correlation coefficient nearing one), the search results in a singular target point. Since rough estimation involves azimuth and elevation angles, a two-dimensional angle estimation is provided. In the first step of the rough estimation, the three targets set are located at the angles corresponding to the same atom. This angle is used to offer a rough location of the three adjacent targets.

**Figure 5:**
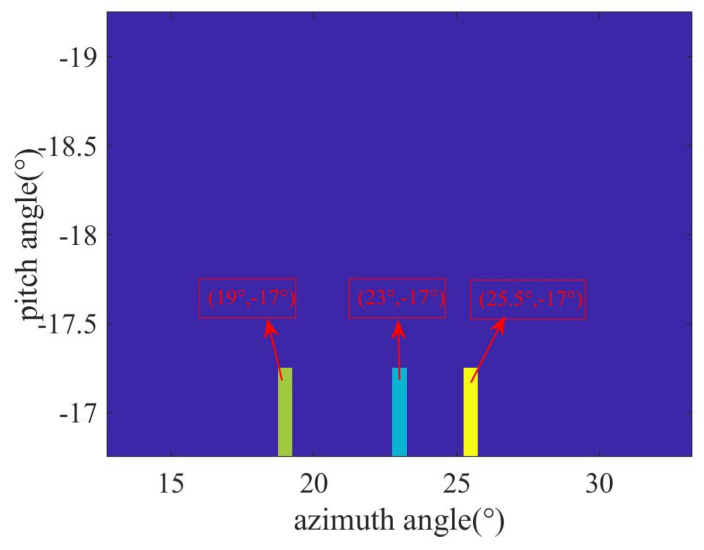
OMP estimation result of refined dictionary.

Using the initial rough estimation ($\varphi_{rough}=24{}^{\circ}$), we plan the trajectory. The half-power beam width corresponding to the synthetic aperture is $\varphi_{hf}=0.015{}^{\circ}$. Consequently, the angular search range for the azimuth angle becomes $\left[\left(\varphi_{rough}-\varphi_{hf}):\Delta \varphi_{rough}/4:(\varphi_{rough}+\varphi_{hf}\right)\right]$, where $\Delta \varphi_{rough}$ represents the azimuth angle search grid during the initial estimation. Since the target elevation angle remains relatively consistent throughout the search phase, the search range for the elevation angle is set at $\left[\left(\theta_{rough}-1{}^{\circ}):\Delta \theta_{rough}/4:(\theta_{rough}+1{}^{\circ}\right)\right]$. We then construct a dictionary matrix corresponding to this refined angle based on the established azimuth and elevation angle grid. Figure 5 displays the refined estimation result. It is evident that all three targets have an estimated elevation angle of −17°, and their estimated azimuth angles are 19°, 23°, and 25.5°, respectively. While the exact azimuth angles of the targets are not pinpointed, the possibility of estimating the three targets within the same beam range became feasible after multiple spatial samplings and refined angle divisions. As there is a greater focus on azimuth angle information during the search state, the rough estimation of the elevation angle is used for a refined azimuth angle estimation. Compared to the first step’s rough estimation, it is clear that the three targets can be distinguished, but the angle values are still not accurate.

**Figure 6:**
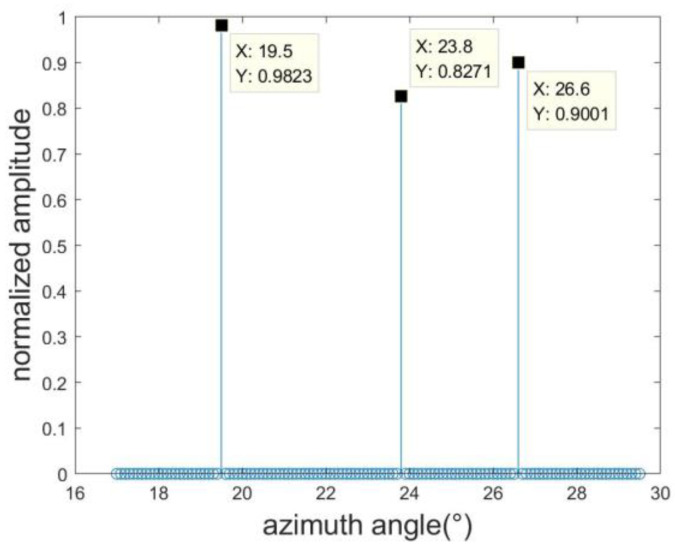
OMP-RELAX estimation result.

To further enhance estimation precision, we employ the RELAX algorithm to fine-tune the target azimuth estimation further. Following the RELAX algorithm’s methodology, when estimating the kth target, the center of the previous estimation result $\varphi_{k}$ is utilized to construct the refined angle search grid $\left[\left(\varphi_{k}-\Delta \varphi_{rough}/4):\Delta \varphi_{rough}:(\varphi_{k}+\Delta \varphi_{rough}/4\right)\right]$ for the kth target. In our study, we define $\Delta \varphi_{rough}=0.1{}^{\circ}$, with the resulting estimations illustrated in Figure 6. The azimuth angles for the three targets are determined to be 19.5°, 23.8°, and 26.6°, which signifies a marked improvement in estimation accuracy compared to the results of the OMP algorithm. Figure 6, based on the elevation angle estimation result of Figure 4 and the azimuth angle estimation result of Figure 5, re-divides the azimuth grid. The results indicate that after processing with the OMP-RELAX algorithm, the estimation accuracy is further improved.

**Figure 7:**
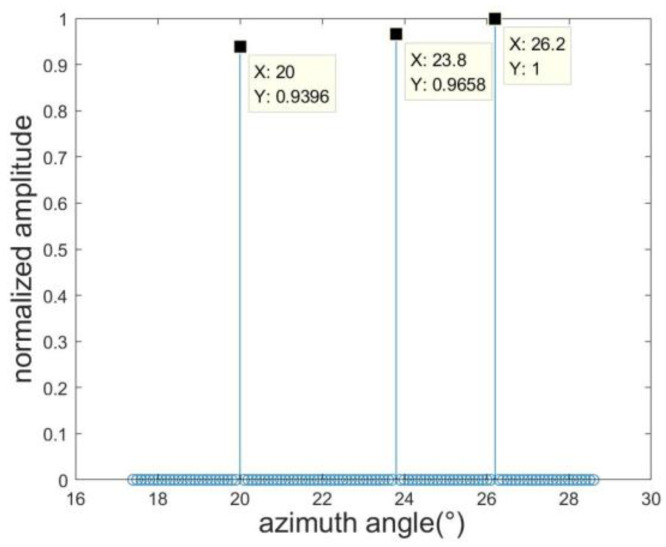
SBL estimation result.

Acknowledging that a target might not align with the precise grid during sparse reconstruction, this study introduces a sequential SBL technique to address the off-grid issue. The outcome of this method is presented in Figure 7. In comparison to the actual target value; the error margin is a mere 0.1°. Collectively, these experiments underscore that our proposed method boasts commendable estimation accuracy. Figure 7, based on the angle estimation of Figure 6, displays the estimation results processed by the SBL algorithm. It is evident from the figure that the estimation result is almost consistent with the target angle.

### 3.2. Robustness Analysis

In this section, we delve into the robustness of the proposed method by assessing its efficacy under varying conditions such as target state (flickering and non-flickering), signal-to-noise ratio (SNR), heading angle, spatial sampling, and target distance. The angle measurement performance for flickering and non-flickering targets is gauged via 1000 Monte Carlo simulations, utilizing the root mean square error (RMSE) of both target azimuth angle and amplitude to measure accuracy. RMSE is defined as

(31) $$\begin{array}{l} RMSE_{DOA}=\sqrt{\frac{1}{MK}\sum_{k=1}^{K}\sum_{m=1}^{M}{\left\|x_{k}-{\hat{x}}_{k,m}\right\|}_{2}^{2}}\\ RMSE_{Amp}=\sqrt{\frac{1}{MK}\sum_{k=1}^{K}\sum_{m=1}^{M}{\left\|\Theta_{k}-{\hat{\Theta }}_{k,m}\right\|}_{2}^{2}} \end{array},$$

where M denotes the number of Monte Carlo trials and K represents the number of targets.

#### 3.2.1. Algorithm’s Adaptability to Various Target States

**Figure 8:**
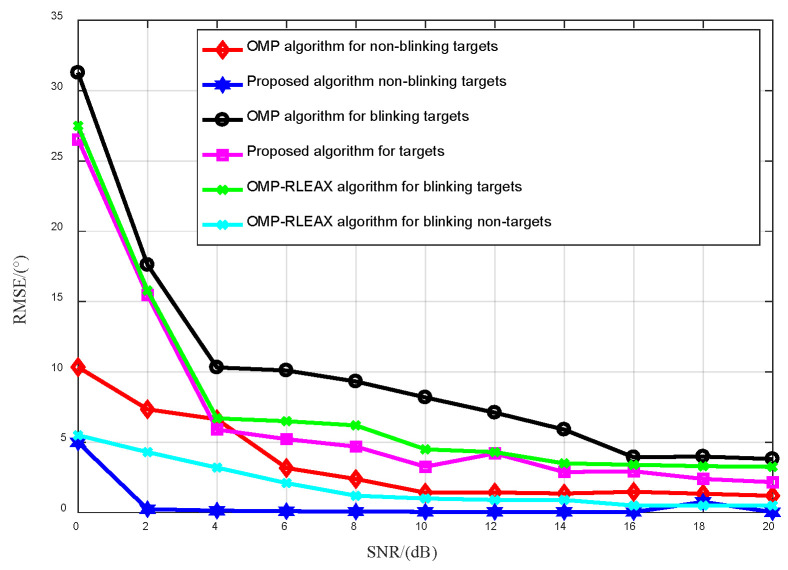
Comparison of angle estimation for flickering and non-flickering targets.

Figure 8 presents a comparative analysis of angle estimation results for diverse target types using the proposed method and other algorithms over a range of SNRs (0~20 dB). In the experiment, evaluations are conducted at intervals of 20 points, totaling 50 evaluations. Observations indicate that our method exhibits superior robustness, especially at lower SNRs, in comparison to alternative algorithms. Although flickering targets can be estimated; they demand a higher SNR than non-flickering targets. This is attributed to the inability of flickering targets to fully accumulate energy during spatial sampling, which subsequently leads to a loss of SNR at specific spatial sampling points. Moreover, the method’s precision in measuring both target types is notable, stemming from three pivotal reasons:

- The OMP-RELAX process minimizes non-orthogonal atom interference.
- The introduced algorithm mitigates the off-grid problem.
- Spatial sampling dynamically refines the dictionary, superseding the original fixed angle grid.

**Figure 9:**
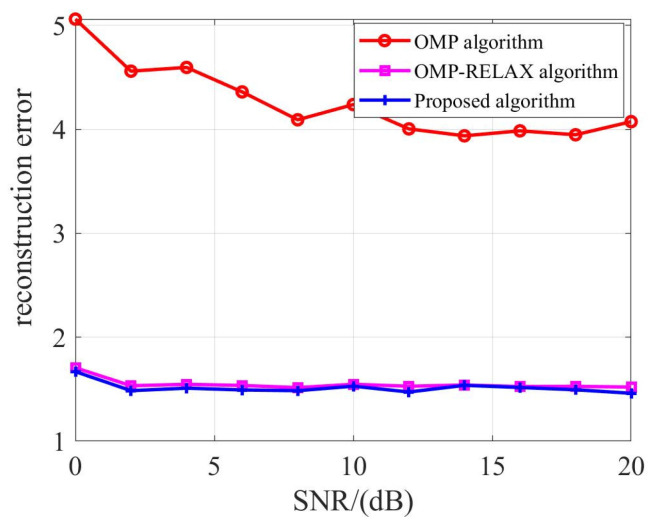
Reconstruction error of a non-flickering target.

The amplitude reconstruction error is depicted in Figure 9. We focus our amplitude estimation exclusively on non-flickering targets for two primary reasons. Firstly, the estimation performance for non-flickering targets surpasses that of flickering targets. Secondly, and more crucially, the inherent unpredictability of target flickering hampers the acquisition of precise amplitude estimation results. The proposed method demonstrates superior amplitude estimation accuracy compared to using OMP alone. This enhanced accuracy can be attributed to two main factors:

- The sequential Bayesian method, as introduced in this study, thoroughly integrates prior information concerning noise and target amplitude.
- Building upon OMP-RELAX, the off-grid Bayesian estimation addresses the issue of position deviation arising from the true angle not aligning with the grid point, thereby bolstering estimation precision.

Figure 9 presents the reconstruction amplitude error for non-flickering targets. The error remains consistent whether the data undergo three (OMP- > MMV-OMP- > OMP-RELAX) or four (OMP- > MMV-OMP- > OMP-RELAX- > SBL) processing steps. This consistency is attributed to the minor angle estimation differences between the three and four-step processes for non-flickering targets. Both are in close proximity to the actual target position, resulting in similar amplitude values.

#### 3.2.2. Impact of Heading Angle on DOA Estimation Accuracy

**Figure 10:**
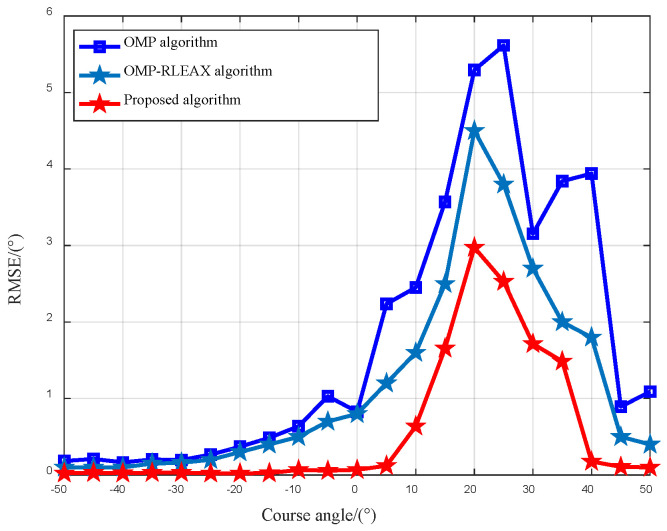
RMSE results across different heading angles.

In this section, our evaluation focuses on non-flickering targets. We consider a heading angle range of $\left[-50{}^{\circ}:5:50{}^{\circ}\right]$, with an SNR set at 20 dB. We conduct 200 Monte Carlo simulations for each specified heading angle, the results of which are depicted in Figure 10.

As can be seen from the figure, under different heading conditions, the proposed method has higher angle measurement accuracy than the OMP algorithm. The target azimuth angle is $\left(\varphi_{1},\varphi_{2},\varphi_{3})\in [20{}^{\circ},30{}^{\circ}\right]$ for the proposed method, and the OMP algorithm in the heading angle range [10°,40°] has a significantly lower angle measurement accuracy compared to other heading angles, with RMSE reaching its peak at heading angles of 20° and 25°. The reason for this phenomenon is that when the heading angle is close to the target angle, the spatial sampling of the aircraft cannot form an effective aperture, so the angle resolution does not improve.

#### 3.2.3. Impact of the Number of Sampling Points on DOA Estimation Results

Sparse reconstruction theory posits that the mutual correlation between dictionary atoms plays a pivotal role in shaping the reconstruction results. Specifically, a diminished mutual correlation is conducive to enhanced reconstruction results. In line with the dictionary design framework proposed in this paper, it is observed that the number of sampling points directly influences the mutual correlation within the dictionary. Hence, we assess the impact of varied sampling point numbers on measurement results by examining the mutual correlation coefficients associated with dictionaries of different sampling sizes.

**Figure 11:**
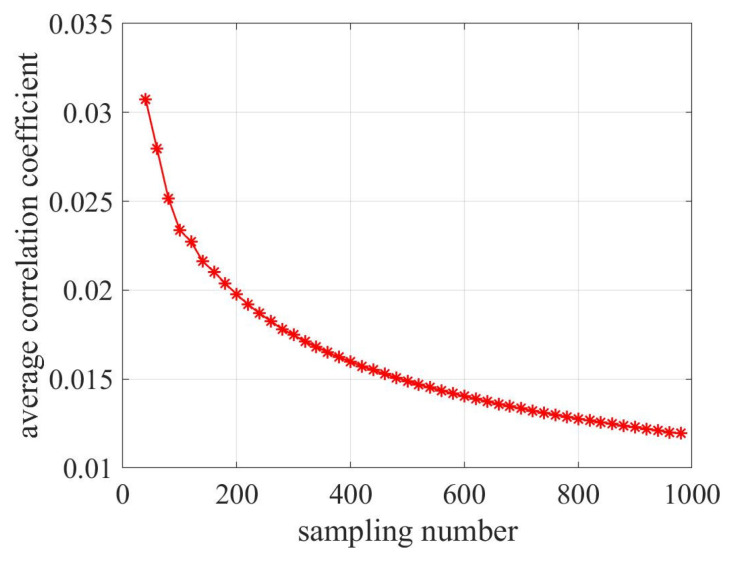
Mean correlation coefficients of the dictionary across varied spatial sampling points.

For the analysis, the aircraft’s maximum spatial sampling points are capped at 1000, maintaining an SNR of 20 dB and a heading angle of −60°. Figure 11 visually represents the mean correlation coefficients of the dictionary matrix across distinct sampling points.

From the figure, a discernible trend emerges. As we increment the number of spatial sampling points, there is a concomitant decline in the dictionary matrix’s average correlation coefficient. This suggests an ongoing enhancement in reconstruction performance. Put another way, with the surge in the count of spatial sampling points, there is an expansion in the spatial synthetic aperture, a contraction in the beam width, and a bolstering of the angular resolution prowess.

#### 3.2.4. Impact of Target Distance on DOA Estimation Accuracy

**Figure 12:**
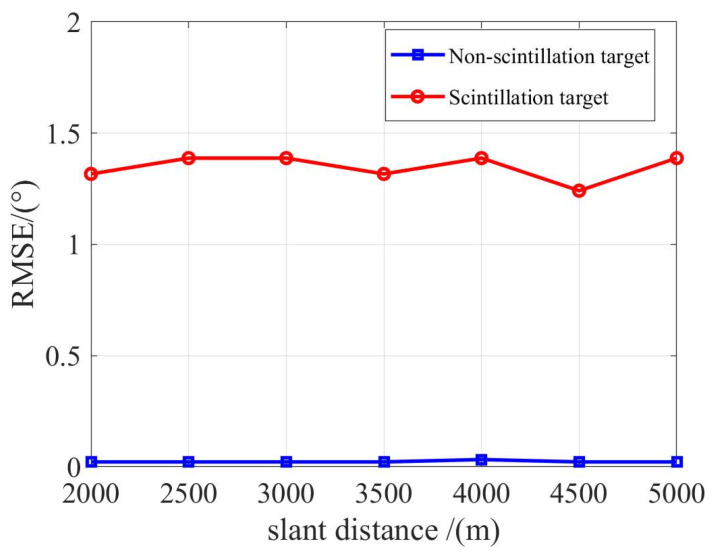
Angle measurement precision for targets across different distances.

To assess how the distance between the aircraft and the target affects angle measurement precision, we examine two distinct scenarios: those involving flickering targets and those with non-flickering targets. Figure 12 illustrates the angle measurement precision for targets at varying distances.

From the data presented in the figure, it is evident that even as the distance to the target extends, there is negligible variation in the angle measurement accuracy for both categories of targets—flickering and non-flickering. This observation underscores the robustness of the proposed algorithm with respect to varying target distances.

### 3.3. Field Experiment

To validate the efficacy of the algorithm, we test the proposed method using real- data. The parameters of the radar system align with those used in the simulation. Additionally, the environment surrounding the radar is characterized by a hilly landscape interspersed with rocks and vegetation, meeting the conditions of a complex terrain.

**Figure 13:**
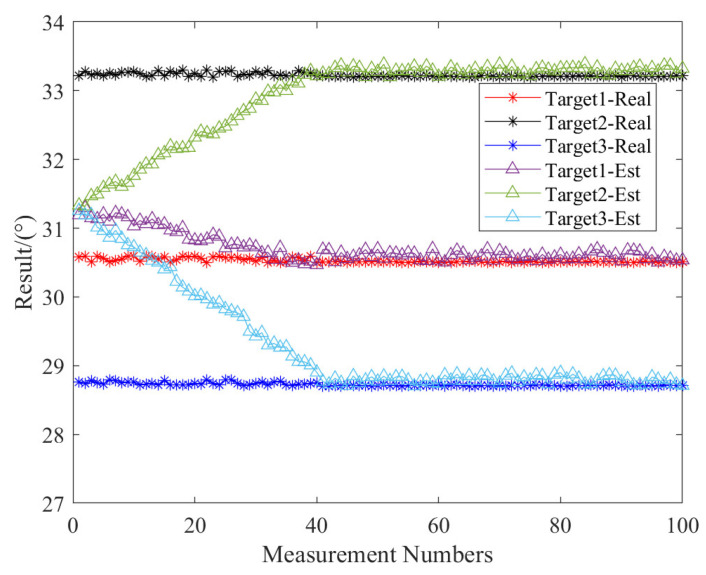
Angle estimation results of the real data by the proposed method.

In our configuration, we set the aircraft’s altitude at 1 km and positioned the radar 5 km from the scene’s center. We arrange three corner reflectors in a triangular pattern on a flat terrain. The aircraft’s actual position is determined using its GPS. Through calculations, we derive the true azimuth angle of the corner reflectors relative to the aircraft. As shown in Figure 13, the star-shaped line represents the true angle values of the three corner reflectors in relation to the aircraft, while the triangular line displays the results of 100 actual measurements. It is clear that during initial sampling, due to a coarse angle estimate, all three reflectors are within the same beam range, exhibiting identical azimuth angles. However, as we increase the measurement count, and consequently the number of spatial sampling points, the spatial synthetic aperture expands, enhancing the accuracy of measurements. By the time we conduct around 40 measurements, we accurately estimate the corner reflectors’ precise azimuth information.

### 3.4. Computational Complexity Analysis

When the original aperture is used to plan the flight direction, the time complexity of the OMP algorithm is *O*(1), a total of four multiplication operations of complex numbers, in which the main computational cost is the inverse operation of *M* × 1 data, where *M* denotes the number of elements. Subsequently, in the process of precise estimation, according to the target number, the MMV-OMP algorithm needs to cycle for *K* times, and so does the OMP-RELAX algorithm, with the time complexity of these two algorithms *O*(*K*) together. The main amount of computation in this stage is the same as that of the OMP algorithm, which comes from the inverse operation of *M* × 1 data. For the off-grid algorithm, assuming that the number of iterations is *Q* and the number of atoms in the dictionary is *P*, the time complexity is *O*(*QP*), a total of (2*Q* + 3*P*) multiplication operations of complex numbers along with the inverse operation of the *T* × *T* matrix as the main computational cost.

Moreover, the single operation time of the OMP algorithm is 0.054 s, the single operation of the MMV-OMP algorithm is 0.006 s, the single operation of the OMP-RLEAX is 0.015 s, the single operation of the off-SBL algorithm is 1.221 s. In the calculation, only one OMP rough estimation is contained in the entire algorithm; then, the precise estimation is carried out on the basis of the rough estimation. On the whole, the calculation time of the proposed algorithm is 1.4 s per process. What is more, the hardware configuration of the computer is CPU·of Intel(R) Core (TM) 7-4770HOCPU @ 2.2 GHz:2.19 GHz, and the memory is 8 GB. In conclusion, although three different algorithms are designed in this paper, only a small number of cycles are required in the calculation process of each algorithm, and high-dimensional data operation is not involved. Thus, the proposed method can ensure the requirement of fast operation. Furthermore, according to the actual measurement result, the estimation accuracy is significantly improved compared with the single goniometric algorithm.

## 4. Conclusions

This paper addresses the issue of limited array aperture DOA estimation performance, which falls short in satisfying the requirements for precise detection. Our approach unfolds as follows:

- Spatial Synthetic Aperture Model Introduction: We present a uniform motion spatial synthetic aperture model. Under this paradigm, the radar enhances DOA estimation accuracy by forming a spatial synthetic aperture through spatial sampling.
- Algorithm Design for High-Precision DOA Estimation: To fulfill both accuracy and real-time demands, we introduce an algorithm that initially employs OMP (Orthogonal Matching Pursuit) for rough target angle estimation and trajectory planning. This algorithm combines the roughly determined target angle with the aircraft’s spatial samples. A measurement dictionary, built upon these spatial samples, then utilizes both OMP-RELAX and sequential off-grid Bayesian techniques for a precise estimation of the spatial sampling data.

Our method offers three distinct advantages over conventional techniques:

- Enhanced Precision: The spatially synthesized array boasts a substantially enlarged aperture and heightened resolution. Our sequential estimation process, paired with Bayesian proportion parameter estimation and dictionary refinement, yields more precise DOA data.
- Increased Efficiency: By employing the greedy method of sparsity reconstruction in DOA estimation, our approach ensures rapid estimations.
- Angle Glint Issues Resolution: The spatial synthetic array sampling and target angle reconstruction can be viewed as a spatial matched filter. Even if the target’s power is diminished over a specific duration, DOA estimation remains feasible through spatial accumulation across an extended period.

In conclusion, the efficacy of our proposed algorithm is substantiated using computer simulation data. The experimental design comprehensively considers variables like target state (both flickering and non-flickering), heading angle, target distance, and evaluates their influence on algorithm precision. While this study focuses on a uniform linear motion model, we do not explore the potential effects of flight trajectory errors on algorithmic accuracy—an avenue we aim to delve into in future research.

## Data Availability

The data presented in this study are available on request from the corresponding author after obtaining permission of an authorized person.

## References

[1] Ma J.J., Ma H., Liu H.W., Liu W., Cheng X.Z. (2023). A novel DOA estimation for low-elevation target method based on multiscattering center equivalent model. IEEE Geosci. Remote Sens. Lett..

[2] Dai X.R., Zhang X.F., Wang Y.F. (2019). Extended DOA-matrix method for DOA estimation via two parallel linear arrays. IEEE Commun. Lett..

[3] Liu Y., Liu H.W., Xia X.G., Zhang L., Jiu B. (2018). Projection techniques for altitude estimation over complex multipath condition-based VHF radar. IEEE J. Sel. Top. Appl. Earth Obs. Remote Sens..

[4] Shi Z., Zhang X.F., Xu L. (2019). DOA estimation of multiple sources for a moving array in the presence of phase noise. IET Signal Process..

[5] Liu G.H., Chen H., Sun X.Y., Qiu R.C. (2016). Modified MUSIC algorithm for DOA estimation with Nyström approximation. IEEE Sens. J..

[6] Stoica P., Nehorai A. (1989). Music, maximum likelihood and Cramer-Rao bound. IEEE Trans. Acoust. Speech Signal Process..

[7] Lee J., Park L., Chun J. (2019). Weighted two-dimensional root MUSIC for joint angle-Doppler estimation with MIMO radar. IEEE Trans. Aerosp. Electron. Syst..

[8] Liu Z.Y., Wu J.Y., Yang S.Y., Lu W. (2022). DOA estimation method based on EMD and MUSIC for mutual interference in FMCW automotive radars. IEEE Geosci. Remote Sens. Lett..

[9] Roy R., Kailath T. (1990). ESPRIT-estimation of signal parameters via rotational invariance techniques. Opt. Eng..

[10] Li J.F., Jiang D.F., Zhang X.F. (2017). DOA estimation based on combined unitary ESPRIT for coprime MIMO radar. IEEE Commun. Lett..

[11] Wang S.H., Cao Y.H., Liu Y.T. (2022). A method of Robust low-angle target height and compound reflection coefficient joint estimation. J. Syst. Eng. Electron..

[12] Chen S., Zhao Y.B., Hu Y.L., Cao C.H., Pan X.J. (2022). Target height and multipath attenuation joint estimation with complex scenarios for very high frequency radar. Front. Inf. Technol. Electron. Eng..

[13] Li J., Sadler B., Viberg M. (2011). Sensor array and multichannel signal processing. IEEE Signal Process. Mag..

[14] Zoltowski M., Haber F. (1986). A vector space approach to direction finding in a coherent multipath environment. IEEE Trans. Antennas Propag..

[15] Zhang X., Liu X.M., Yu H.X. (2015). Improved MUSIC algorithm for DOA estimation of coherent signals via Toeplitz and fourth-order-cumulants. Int. J. Control Autom..

[16] Barcelo M., Vicario J.L., Seco-Granados G. (2011). A reduced complexity approach to IAA beamforming for efficient DOA estimation of coherent sources. Eurasip J. Adv. Signal Process..

[17] Yuan X. (2014). Coherent sources direction finding and polarization estimation with various compositions of spatially spread polarized antenna arrays. Signal Process..

[18] Wang Q., Chen H., Zhao G.H., Chen B., Wang P.C. (2013). An improved direction finding algorithm based on Toeplitz approximation. Sensors.

[19] Hyder M.M., Mahata K. (2010). Direction-of-Arrival estimation using a mixed L2 norm approximation. IEEE Trans. Signal Process..

[20] Wen J., Liao B., Guo C.T. (2017). Spatial smoothing based methods for direction-of-arrival estimation of coherent signals in nonuniform noise. Digit. Signal Process..

[21] Qi C.Y., Wang Y.L., Zhang Y.S., Han Y. (2005). Spatial difference smoothing for DOA estimation of coherent signals. IEEE Signal Process. Lett..

[22] Jiang G.J., Yang Y.L. (2023). Synthetic sparse nested array with extended aperture and reduced mutual coupling for DOA estimation. Signal Process..

[23] Gorodnitsky I.F., Rao B.D. (1997). Sparse signal reconstruction from limited data using FOCUSS: A re-weighted minimum norm algorithm. IEEE Trans. Signal Process..

[24] Wang J., Kwon S., Shim B. (2012). Generalized orthogonal matching pursuit. IEEE Trans. Signal Process..

[25] Gerstoft P., Mecklenbräuker C., Xenaki A., Nannuru S. (2016). Multisnapshot sparse Bayesian learning for DOA. IEEE Signal Process. Lett..

[26] Chen J., Huo X.M. (2006). Theoretical results on sparse representations of multiple-measurement vectors. IEEE Trans. Signal Process..

[27] Zhuang S.Y., Zhao W., Wang Q., Wang Z., Chen L., Huang S.L. (2019). A high-resolution algorithm for supraharmonic analysis based on multiple measurement vectors and Bayesian compressive sensing. Energies.

[28] Herzet C., Drémeau A., Soussen C. (2016). Relaxed recovery conditions for OMP/OLS by exploiting both coherence and decay. IEEE Trans. Inf. Theory.

[29] Yang Z., Xie L.H., Zhang C.S. (2012). Off-grid direction of arrival estimation using sparse Bayesian inference. IEEE Trans. Signal Process..

[30] Chen F.H., Dai J.S., Hu N., Ye Z.F. (2018). Sparse Bayesian learning for off-grid DOA estimation with nested arrays. Digit. Signal Process..

[31] Lin J.C., Ma X.C., Yan S.F., Jiang L. (2015). Pattern synthesis of sparse linear array by off-grid Bayesian compressive sampling. Electron. Lett..

